# Uptake of intermittent preventive treatment of malaria in pregnancy and risk factors for maternal anaemia and low birthweight among HIV-negative mothers in Dschang, West region of Cameroon: a cross sectional study

**DOI:** 10.1186/s12936-023-04816-8

**Published:** 2024-01-04

**Authors:** Sabrina Lynda Simo Kamga, Innocent Mbulli Ali, Ghislain Romeo Ngangnang, Mehmet Can Ulucesme, Leonard T. D. Keptcheu, Eva Mai Keming, Valery-Pacome Kom Tchuenkam, Juluis Visnel Foyet, Münir Aktas, Michel Noubom, Vincent K. Payne

**Affiliations:** 1 Research Unit of Biology and Applied Ecology, Department of Animal Biology, Faculty of Science, University of Dschang, Dschang, Cameroon; 2 Research Unit of Microbiology and Antimicrobial Substances, Department of Biochemistry, Faculty of Science, University of Dschang, Dschang, Cameroon; 3 Department of Clinical Biology, Faculty of Medicine and Pharmaceutical Sciences, University of Dschang, Dschang, Cameroon; 4 The Biotechnology Centre, University of Yaounde 1, Yaounde, Cameroon; 5 Laboratory of Molecular Parasitology, Department of Parasitology, University of Firät, Elazig, Turkey

**Keywords:** IPT, Birth weight, Maternal anemia, Malaria, Pregnancy, Dschang

## Abstract

**Background:**

Approximately 32 million pregnant women are at risk of malaria with up to 10,000 maternal deaths and 200,000 neonates at risk annually. Intermittent Preventive Treatment (IPT) with sulfadoxine-pyrimethamine (SP) is recommended by the World Health Organization (WHO) to reduce disease in pregnancy and adverse maternal and newborn outcomes. At least three doses of SP should be taken by pregnant women during antenatal consultation (ANC) beginning from the thirteenth week of pregnancy till parturition. The aim of this study was to assess uptake of IPT during pregnancy and risk factors for maternal anaemia and infant birth weight in Dschang, West region of Cameroon.

**Methods:**

A total of 380 consenting pregnant women at delivery were recruited in a cross- sectional prospective survey between January to December 2021. Data on ANC attendance, total dose of IPT and history of malaria were abstracted from hospital ANC records while socio-demographic characteristics, bed net use and obstetrics history of each participant were also recorded through an interview. Further, blood samples were collected from the intervillous space for assessment of maternal anaemia and microscopic parasitology. Nested PCR based on amplification of the *Plasmodium* 18S sRNA was carried out to detect submicroscopic infection. IPTp coverage was calculated per WHO recommendation and the prevalence of anaemia and low birth weight were estimated as proportions in the total sample of pregnant women and live births, respectively. Crude and adjusted odds ratios and their 95% confidence intervals were used to estimate associations between pregnancy outcomes considered and risk factors in specific and general models. A p < 0.05 was considered significant. The R software (V4.1.4) was used for all analyses.

**Results:**

A majority of pregnant women was aged between 24 and 34 years old (59.2%) and had secondary education (58.8%). Uptake of ≥ 3 IPTp was 64.99% with 77.20% of all who received at least one IPTp doses taking a mix of SP and DP or DP alone in successive ANC contacts. Those with four or more ANC contacts (73.42%) were more likely to have received at least one IPTp. Furthermore, 13.9% of live births had low birthweights (BW < 2500 *g*) and one in four parturient women with moderate anaemia by WHO criteria. Microscopy (blood smear examination) and PCR-based diagnosis revealed between 0% and 1.57% of parasite-infected placental samples, respectively. Reported malaria in pregnancy predicted maternal anaemia at birth but not birth weight. Only gestational age (< 37 weeks) and bed net use (< 5 months) significantly predicted infant birth weight at delivery.

**Conclusion:**

The uptake of WHO recommended IPT doses during pregnancy was moderately high. Reported malaria in pregnancy, poor bed net coverage, gestational age less than 37 weeks adversely affect maternal haemoglobin levels at birth and infant birth weight. Asymptomatic and submicroscopic placental parasite infections was found at low prevalence. Together these results highlight the importance of maintaining aggressive measures to prevent malaria in pregnancy and protect the health of mother and baby.

**Supplementary Information:**

The online version contains supplementary material available at 10.1186/s12936-023-04816-8.

## Background

Malaria is a disease caused by an intra-erythrocyte protozoan of the genus *Plasmodium* [1]. These pathogens are transmitted from one person to another through the bites of infected female *Anopheles* mosquitoes or transplacentally by mother to fetus during pregnancy [2]. Malaria is an important public health concern among pregnant women, because it is associated with severe complications for the mother and baby; including maternal anaemia, pregnancy loss and preterm babies, intrauterine growth retardation and perinatal mortality [3–5]; approximately 32 million of pregnant women are at risk worldwide. Five species of *Plasmodium* are responsible for disease in humans, of which *Plasmodium falciparum* is responsible for most of the pathologies in pregnancy [6, 7]. The 2022 World Malaria Report, described more than 13 million cases of malaria in pregnancy globally [8], and one year earlier, the prevalence of malaria in pregnancy in Cameroon had been reported to be 39.8% [9].

Among pregnant women living in stable malaria transmission areas, few infections lead to symptomatic malaria. However, malaria infections in asymptomatics cause maternal morbidity, such as anaemia, and adverse pregnancy effects, such as abortion, low birth weight and infant mortality [10]. In low transmission areas of malaria, where women of child-bearing age have no acquired immunity to malaria, malaria correlates with anaemia, causes severe acute syndromes in mothers, increased risk of severity including abortion and low birth weight of babies at delivery and promotes fetal mortality [10]. In areas of high transmission, malaria is shown to be more prevalent among primiparous women and the parasite density decreases with the number of pregnancies [11]. Further, it has been reported that submicroscopic infections are highly prevalent in low malaria transmission settings, and could be responsible for placental pathology and maternal anaemia [12–14].

While there are reports of placental infections in pregnancy in settings of perennial malaria transmission in Cameroon, such as in the forest and southern littoral areas [15–23], there are also reports of malaria in pregnancy and placental infections in the low transmission western highlands of Cameroon, and where *Plasmodium vivax* has been previously described [24]. In an attempt to contribute to a fuller understanding of the epidemiology of placental malaria and submicroscopic infections as well as IPTp coverage under routine implementation, this study aimed to determine the prevalence of placental infections, coverage of IPTp and factors associated with maternal anaemia and infant weight at delivery among HIV-1 negative mothers in Dschang, West region of Cameroon.

## Methods

### Ethics statement

The study was approved by the Institutional Review Board of the Cameroon Baptist Convention Health Services through reference number IRB2019-38, whereas administrative authorization was obtained from the West Regional Delegation of Public Health. Written informed consent was obtained from all the study participants prior to data and sample collection.

### Study site

**Fig. 1:**
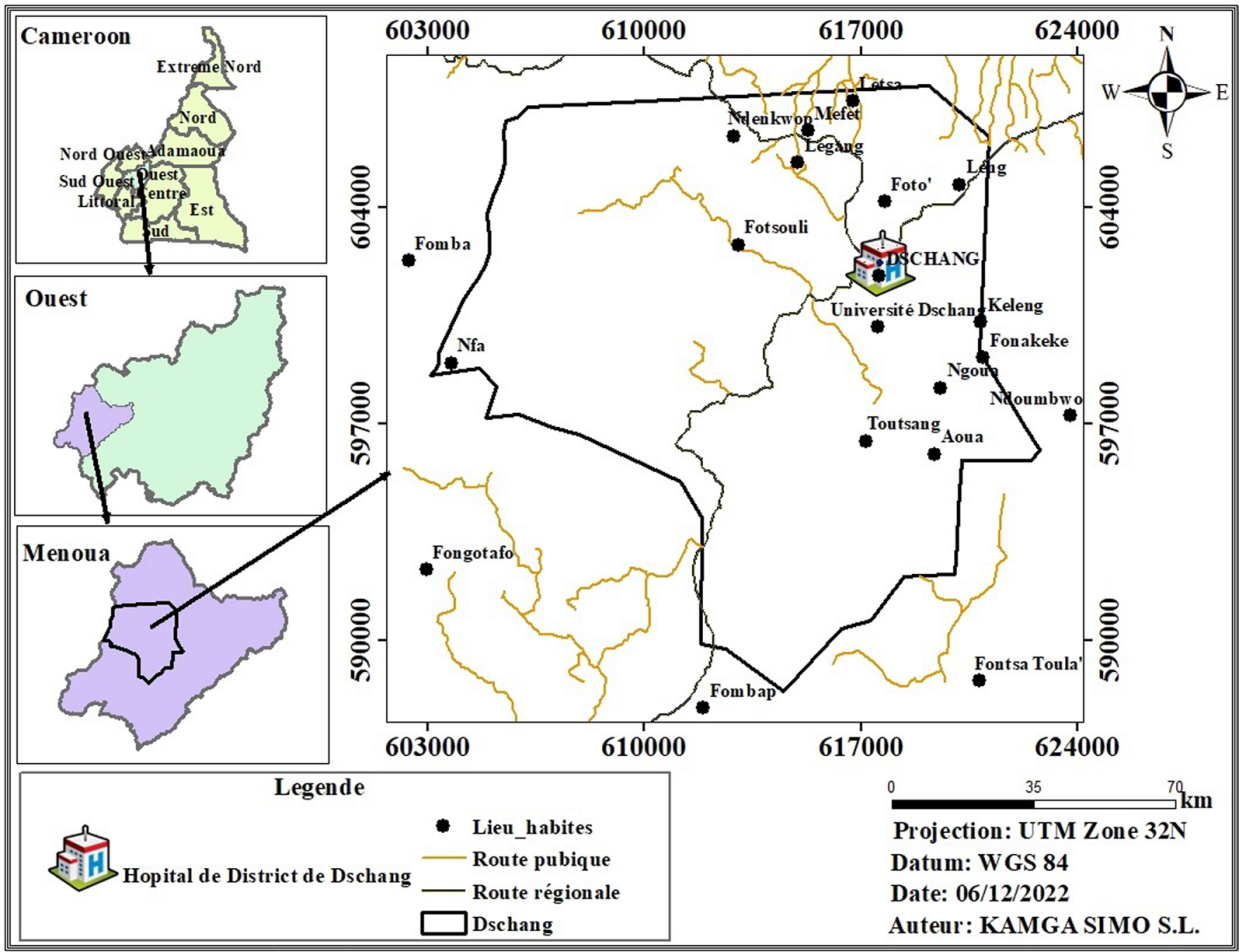
A map showing the location of the Dschang District Hospital where the study took place

The study was conducted at the maternity unit of the Dschang Regional Hospital Annex (Fig. 1) located in Dschang, West Region of Cameroon from January to December 2021. The Menoua division is made of five health districts for which the Dschang Health District is the largest. It is located between 5°25ʹ–5°30 of North and 10°–10°5ʹ East at 213 km North of Douala and 350 km North-West of Yaoundé, the capital of Cameroon. This city is bound on the southern side by the Bamboutos Mountains and in the southwest by the Menoua River flowing to the Mbo plain. In the East, it is bound by the Bani Mountain that culminates at more than 1920 m. The town registers more than 1900 mm precipitation per year with an average temperature of 20.2 ℃. Geographically, Dschang lies between the altitude savannah and the mountain forest and is covered with very dense vegetation [25]. Dschang Regional Hospital Annex was selected for this study because it is the main public hospital that provides accessible and affordable antenatal care which allows enrolment of the majority of pregnant women at delivery reflecting the profile in the study area.

### Study design and population

Consenting pregnant women were enrolled consecutively through a cross-sectional survey at delivery in the maternity. Enrolment by the time of delivery ensured uptake of adequate doses of IPT as stipulated by the World Health Organization (WHO). Mothers with evidence of AIDS were not eligible for the study. Pregnant women were enrolled if they attended at least one ANC visit at the district hospital, did not have complications, such as hypertension or other conditions, that required emergency care. The target population had to provide a written informed consent to study procedures including consent to publish de-identified study results in different media, including in peer reviewed scientific journals. The sample size was calculated using the formula below. It was based on the

$${\text{n }} = \, {{\left[ {{\text{DEFF}}*{\text{Np}}\left( {{1} - {\text{p}}} \right)} \right]} \mathord{\left/ {\vphantom {{\left[ {{\text{DEFF}}*{\text{Np}}\left( {{1} - {\text{p}}} \right)} \right]} {\left[ {\left( {{\text{d2}}/{\text{Z21}} - \alpha /{2}*\left( {{\text{N}} - {1}} \right) + {\text{p}}*\left( {{1} - {\text{p}}} \right)} \right)} \right]}}} \right. \kern-0pt} {\left[ {\left( {{\text{d2}}/{\text{Z21}} - \alpha /{2}*\left( {{\text{N}} - {1}} \right) + {\text{p}}*\left( {{1} - {\text{p}}} \right)} \right)} \right]}}$$

where = 38.5% based on analysis of the 2018 demographic health survey data in Cameroon [26]. A sample of 364 was obtained, which was adjusted by a 4.5% rate of refusal to reach a final minimum sample of 380 pregnant women at delivery.

### Data and samples collection

A semi-questionnaire was designed pretested and further used to obtain data related to maternal, obstetric and socio-demographics, antenatal care history, malaria during pregnancy, intake of IPT. ANC records were used to abstract missing information or verify reported exposure to IPT and malaria during pregnancy and maternity registers for obstetrics history. The weight of the newborn was measured using a sensitive baby scale. The average of two readings was recorded as the birth weight. Weight scales were within the period of warranty for weight accuracy per the manufacturer. Data on the type of medication used for IPT as well as any treatment for malaria infection during pregnancy was also collected.

About 2 mL of venous blood from consenting pregnant women which was used to measure haemoglobin (Hb) concentration using a hand-held haemoglobinometer (HemoCue, Angelholm, Sweden) according to the manufacturer’s instructions. Anaemia status was defined based on WHO criteria as Hb concentration less than 11.0 *g*/dL and classified based on severity as mild (Hb levels 9 to 10.9 *g*/dL), moderate (Hb levels 7 to 8.9 *g*/dL), and severe (Hb levels less than 7 *g*/dL) [27]. Dried blood spots, thick and thin blood smears were also prepared from the intervillous space of the placenta after the latter was expelled. Midwives assisted in handling the placenta. Blood from the intervillous space was collected by placental prick which accessed intervillous blood through the chorionic plate. The blood was used for microscopy and PCR-based diagnosis.

Malaria microscopy was performed using standard WHO procedures [28]. The smears were stained with 10% Giemsa for 15 min and then read under a binocular light microscope using × 100 objective and immersion oil by two microscopists at different times. The microscopist were blinded to the each other's results. In the case of discrepancy (such as presence of parasite in thick blood smear by one technician and absence by another) noted by the investigator, a more senior technician was asked to re-read the slide. The criteria for malaria parasite positivity used by microscopists during observation was the detection of asexual stages of the parasite (rings, trophozoites or schizonts) in the thick film, the identification of any parasite species in the thin film, and if positive to determine the parasite density in the thick film using the standard formula considering 8000 leucocytes present per microlitre of blood. In case of low parasite density, the protocol was that the reading time will be prolonged to include counting parasites against at least 500 leucocytes before validating the results.

### Molecular detection of parasite infection

2–5 pieces (2–3 mm) of dried blood drops were cut from Whatman FTA cards and placed in sterile microcentrifuge tubes. Firstly, 100 μL phosphate buffered saline (PBS) and 20 μL Proteinase-K were added to the tube. It was mixed well by vortexing and incubated for 30 min at 55 ℃ in a water bath. Then, 180 μL genomic digestion buffer was added to the tube and it was incubated again at 55 ℃ for one and a half hours. After this step, genomic DNA extraction was extracted by a commercial DNA isolation kit (Invitrogen Corporation, Carlsbad, CA, USA) following the manufacturer’s instructions.

To detect positive samples, a nested PCR test was performed using two genus-specific primers pairs amplifying the 18S ribosomal RNA (rRNA) gene region of the *Plasmodium* spp. For initial amplification of and 1670 bp fragment of the 18S rRNA gene, rPLU1 (5ʹ-TCAAAGATTAAGCCATGCAAGTGA-3ʹ) and rPLU5 (5ʹ CCTGTTGTTGCCTTAAACTCC-3ʹ) primers were used. The nested amplification was performed using the rPLU3 (5ʹ- TTTTTATAAGGATAACTACGGAAAAGCTGT-3ʹ) and rPLU4 (5ʹ- TACCCGTCATAGCCATGTTAGGCCAATACC-3ʹ) primers, to amplify a fragment 235 bp. Nested PCR was performed to detect *Plasmodium falciparum* in all samples. For this purpose, the first PCR products were used as template and amplified using rFaL1 (5ʹ-TTAAACTGGTTTGGGAAAACCAAATATATT-3ʹ)/rFaL2 (5ʹ- ACACAATGAACTCAATCATGACTACCCGTC-3ʹ) primers, which amplified the 206 bp fragment of the 18S rRNA gene [29]. The PCR was performed in a total reaction volume of 25 μL containing 2.5 μL of 10 × PCR buffer [100 mM Tris–HCl (pH 9), 500 mM KCl, 1% Triton X-100], 250 μM of each of the four deoxynucleotide triphosphates, 2 U Taq DNA polymerase (Promega, Madison, WI, USA), and 10 pmol of each primer. The PCR reaction conditions are detailed in Additional file 1. The amplicons were analysed by 1.5% agarose gel electrophoresis and visualized using the gel documentation system (Vilber Lourmat, Marne La Vallee Cedex, France).

### Data analysis

The primary outcomes for the current study were uptake of IPTp during pregnancy, and infant birth weight and maternal anaemia. Secondary outcomes were the prevalence of placental malaria parasite infection, and determinants of anaemia and low birth weight in the study population. Anaemia was defined according to WHO as Hb concentration less than 11.0 *g*/dL and classified based on severity as mild (Hb levels 9 to 10.9 *g*/dL), moderate (Hb levels 7 to 8.9 *g*/dL), and severe (Hb levels less than 7 *g*/dL). Infant birth weight was categorized as measured birth weight < 2500 *g* averaged from two measurements which was done for this study by two trained midwives and monitored by one study investigator. Main exposure variable was IPT intake (adherent to recommendations: ≧3 IPT vs. non-adherent: < 3 IPT during pregnancy). Explanatory variables included socio-demographic characteristics, malaria exposure, bed net use (yes/no), duration of bed net use (< 5 months, ≧ 5 months) gestational age (< 37 weeks), parity (< 5), gravidity (≦3, > 3) and level of education (primary, secondary, tertiary), residence (urban, rural), and number of ANC contacts (< 4, ≧4).

Data were transcribed into Microsoft Excel 2010 spreadsheet and verified by a second study technician. Ten percent of entries were monitored from source documents by another investigator to gauge the quality of entries. After data cleaning and resolution of queries, the data sheet was uploaded in R software (R version 4.1.4) for statistical analysis. Generalized linear modelling was used to examine the effect of covariates where IPTp uptake, maternal anaemia and infant low birth weight were entered separately as dependent variables. Maternal age, educational level, bed net use, gestational age, gravidity, parity, number of antenatal care contacts, residence and reported exposure to malaria in pregnancy were explanatory variables. The forward variable selection algorithm was used to examine the effect of each covariate at a time and the best models with the lowest archaic information criterion selected. Test for multicollinearity was also performed by estimating variance inflation factors for the variables which were found to be under 2 for each of the examined variables in the models retained. Models plots showed the adjusted odds ratios of the different variables for each outcome in the final model and were transcribed to a table. A p-value of 0.05 was considered the threshold for significance.

## Results

### Characteristics of study population

Data collected from 380 consenting women are summarized in Table 1 below. These results revealed that participants aged between 24 and 34 years old were the most represented (59.2%) while those aged more than 34 years were the least (11.8%). Moreover, most pregnant women had secondary level of education (56.8%). Of all consenting participants, more than half (54.2%) did not sleep under a mosquito bed net while among those who used it, the majority reported using for than five months (53%). Furthermore, 37.9% of women were primigravidae, while 32.4% did not have children at home. The gestational age at delivery was > 37 weeks for a majority of the women (84.2%). Up to 17.1% of women were febrile at delivery.

**Table 1:** Characteristics of study population

Characteristics	Number	Percentages%
Age groups (years)		
14–23	110	28.9
24–34	225	59.2
> 34	45	11.8
Urbanity		
Semi urban	277	72.89
Urban	103	27.10
Fever status		
Febrile(T℃≧ 37.5)	65	17.1
Afebrile (T℃ < 37.5)	315	82.9
Level of education		
Primary	7	1.84
Secondary	216	56.8
Tertiary	157	41.3
Bed net use		
Yes	181	47.63
No	199	54.37
Duration of bed net use (months)		
≧5	85	46.96
< 5	96	53.03
Number of ANC		
< 3	25	06.58
≧ 3	355	93.42
Gravidity		
1	144	37.89
2	69	18.15
3	72	18.94
> 3	128	33.68
Parity		
0	123	32.36
1	82	21.57
2	75	19.73
3	41	10.78
> 3	59	15.52
Gestational age (weeks)		
≥ 37	348	84.2
< 37	32	15.8
Malaria in pregnancy		
Yes	96	25.3
No	283	74.5
Maternal anaemia		
Anaemia	93	25.00
No anaemia	285	75.00
Intake of IPT		
0	17	4.47
1	41	10.78
2	73	19.21
3	113	29.73
> 3	134	35.26
Type of IPTp drug		
IPTp-SP only	155	40.79
IPTp-DP only	30	07.89
IPTp-SP/DP seq*	179	47.11
No IPTp	16	4.21
Weigh of babies at delivery		
≥ 2500	327	86.1
< 2500	53	13.9

^*^ Intermittent preventive treatment with DP or SP sequentially in subsequent ANC contacts

**Fig. 2:**
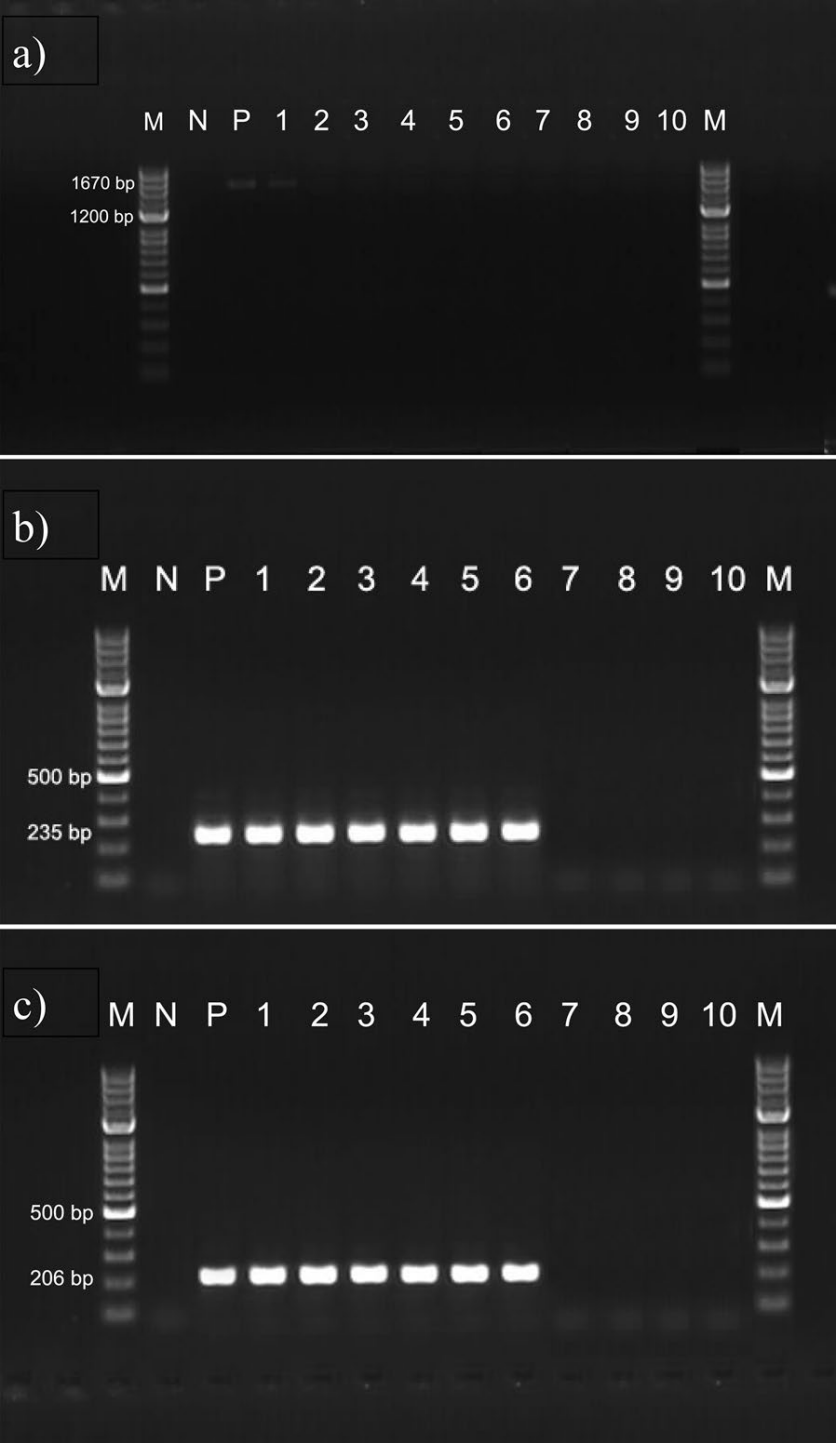
Agarose gel visualization of amplified 18S rRNA gene region of *Plasmodium* spp*.* **a** PCR products of amplified with primers rPLU1/rPLU5 for the *18S rRNA* gene region of *Plasmodium* spp. M, 100 bp ladder; N, negative control (distilled water); P, standard positive controls (*P. falciparum*); lane 1, positive field sample; lanes 2–10, negative field samples. **b** Nested PCR products of amplified with primers rPLU3/rPLU4 for the *18S rRNA* gene region of *Plasmodium* spp. M, 100 bp ladder; N, negative control (distilled water); P, standard positive controls (*P. falciparum*); lanes 1–6, positive field samples; lanes 7–10, negative field samples. **c** Nested PCR products of amplified with primers rFAL1/rFAL2 for the *18S rRNA* gene region of *P. falciparum*. M, 100 bp ladder; N, negative control (distilled water); P, standard positive controls (*P. falciparum*); lanes 1–6, positive field samples; lanes 7–10, negative field samples

The prevalence of malaria in pregnancy (MIP) based on reported infection during pregnancy was 25.3%. At delivery, 24.5% of women presented with anaemia, although severe anaemia was recorded only in 2 (0.53%) women. No parasite was seen upon slide microscopy of intervillous blood smears. However, PCR based diagnosis revealed six placental infections from afebrile women giving an asymptomatic submicroscopic infection prevalence of 1.57% (6/380). Figure 2 shows band positions corresponding to PCR amplification of *Plasmodium* spp DNA fragments in placental blood samples.

It was also noted in this study that 93.4% of pregnant women had at least three antenatal (ANC) contacts prior to delivery. Overall, 13.9% (53/379) of live infants had low birth weight. Only one still birth was recorded and no twins. Sixty-four percent (64.99%) of the women at delivery had taken three or more doses of IPT while 4.2% did not take any chemoprevention during pregnancy. Thirty (7.9%) participants took IPTp-DP during the ANC period while 40.7% were exposed to IPTp-SP. The majority (47.2%) took a mix of both types of drugs in subsequent ANCs.

Uptake of intermittent preventive treatment according to characteristics of the study population.

Table 2 below summarizes the association between IPT uptake and characteristics of study population. IPT uptake of three or more IPT doses was associated only with the number of ANC contacts (p < 0.001) and this association remained significant in multivariate regression analysis. There were no associations observed between IPTp uptake and age group, parity, gravidity, bed net use or the duration of use in the study population.

**Table 2:** Association between sociodemographic, bed net use, gestational and obstetric characteristics and uptake of ≥ 3 IPT doses in the study population

Variables	Modalities	Number (%)	cOR (95% CI)	P	aOR (95% CI)	P
Age group	15—23	37 (33.64)	1	–	1	-
	24—34	75 (33.48)	0.99 (0.61–1.62)	0.98	0.97 (0.49–1.90)	0.92
	> 34	22 (48.89)	1.89 (0.93 3.84)	0.08	2.38 (0.83–7.07)	0.11
Education level	Secondary	71 (32.87)	1	–	1	–
	Primary	4 (57.14)	4.08 (0.782 9.97)	0.11	2.94 (0.43 29.20)	0.30
	Tertiary	59 (37.58)	1.23 (0.80–1.89)	0.35	0.94 (0.57–1.57)	0.82
Bed net use	Yes	63 (36.21)	1	–	1	–
	No	71 (34.47)	0.92 (0.60–1.40)	0.69	0.89 (0.48–1.66)	0.72
Duration of bed net use	≧5	32 (33.68)	1	–	1	–
	< 5	102 (35.79)	1.08 (0.66 – 1.78)	0.76	1.38 (0.67–2.84)	0.38
Gravidity	≧2	99 (37.22)	1	–	1	–
	< 2	35 (30.70)	0.76 (0.47–1.20)	0.24	0.84 (0.43–1.60)	0.59
Parity	≧3	41 (41.00)	1	–	1	–
	< 3	93 (33.21)	0.72 (0.45–1.15)	0.17	0.80 (0.42–1.53)	0.50
Number of ANC contacts	≧4	131 (47.12)	1	–	1	–
	< 4	3 (2.94)	0.03 (0.01–0.09)	< 0.001^***^	0.03 (0.01 – 0.08)	< 0.001^***^
Gestational age	≧37	123 (35.55)	1	–	1	–
	< 37	11 (32.35)	0.86 (0.39–1.79)	0.70	1.33 (0.52–3.37)	0.55
Residence	Semi urban	95 (34.30)	1	–	1	–
	Rural	39 (37.86)	1.19 (0.74–1.89)	0.48	1.04 (0.60–1.79)	0.89
Exposure to malaria	No	92 (34.72)	1	–	1	–
	Yes	42 (36.52)	1.10 (0.69–1.73)	0.69	1.01 (0.60–1.70)	0.97

^**^ Indicates significance results (p < 0.05)

### Relationship between explanatory variables and anaemia in the study population

Table 3 shows the relationship of sociodemographic and obstetric factors with maternal anaemia outcome in the study population. Analysis found that gestational age < 37 weeks marginally associated with maternal anaemia in bivariate models diminished to insignificance in multivariate analysis. Additionally, only reported malaria during pregnancy was significantly associated with maternal anaemia at delivery in both bivariate and multivariate models. No association was found between other characteristics and maternal anaemia at delivery (Table 3).

**Table 3:** Association between socio-demographic and obstetric characteristics and maternal anaemia at delivery in the study sample

Variables	Modalities	Number (%)	cOR (95% CI)	P	aOR (95% CI)	P
Age group	15–23	31 (28.18)	1	–	1	–
	24–34	52 (23.21)	0.77 (0.46–1.30)	0.34	0.71 (0.35 1.44)	0.34
	> 34	11 (24.44)	0.82 (0.36 1.79)	0.63	0.75 (0.26 2.12)	0.59
Education level	Secondary	57 (26.39)	1	–	1	–
	Primary	3 (42.86)	2.09 (0.40 6.77)	0.34	1.25 (0.16 7.39)	0.81
	Tertiary	35 (22.29)	0.80 (0.49–1.29)	0.36	0.85 (0.50 1.45)	0.56
Bed net use	Yes	44 (25.29)	1	–	1	–
	No	51 (24.76)	0.97 (0.61–1.55)	0.91	1.12 (0.60 2.13)	0.72
Number of net use	≧5	22 (23.16)	1	–	1	–
	< 5	73 (25.61)	1.14 (0.67–2.00)	0.63	1.00 (0.47 2.10)	0.99
Gravidity	≧2	68 (25.56)	1	–	1	–
	< 2	27 (23.68)	0.90 (0.54–1.50)	0.70	0.66 (0.33 1.32)	0.25
Parity	≧3	26 (26.00)	1	–	1	–
	< 3	69 (24.64)	0.93 (0.56–1.59)	0.79	0.91 (0.47 1.76)	0.77
Number of ANC contacts	≧4	71 (25.54)	1	–	1	–
	< 4	24 (23.53)	0.90(0.52–1.51)	0.69	0.90 (0.50 1.58)	0.72
Gestational age	≧37	82 (23.70)	1	–	1	–
	< 37	13 (38.24)	1.99 (0.93–4.11)	0.06	1.96 (0.884.25)	0.09
Residence	Semi urban	66 (23.83)	1	–	1	–
	Rural	29 (28.16)	1.25 (0.75–2.07)	0.39	1.05 (0.60 1.81)	0.86
malaria in pregnancy	No	48 (18.11)	1	–	1	–
	Yes	47 (40.87)	3.12 (1.92–5.09)	< 0.001^***^	3.23 (1.94 5.42)	< 0.001^***^

^***^ Indicates significant association (P < 0.05)

### Association between socio-demographic and obstetric factors and birth weight of infants in the study population

The association between low birth weight (LBW) and sociodemographic and obstetrical characteristics of the study population was studied. As Table 4 shows, low birth weight was found to be associated only with gestational age less than 37 weeks. Further, it was noted that among infants with low birth weight, the most represented were born to nulliparous mothers, aged between 24 and 34 years who did not use a bed net and had taken less than 3 doses of IPT, although the relationship between these variables was not significant.

**Table 4:** Association between maternal and sociodemographic and obstetric factors and Low birth weight infants (< 2500 *g*) in the study population

Variables	Modalities	Number (%)	cOR (95% CI)	P	aOR (95% CI)	P
Age group	15–23	19 (17.27)	1	–	1	–
	24–34	30 (13.39)	0.74 (0.40–1.40)	0.35	0.97 (0.40–2.37)	0.95
	> 34	2 (4.44)	0.22 (0.03–0.81)	0.05^*^	0.25 (0.03–1.31)	0.13
Education level	Secondary	25 (11.57)	1	–	1	–
	Primary	1 (14.29)	1.53 (0.08–9.99)	0.70	2.71 (0.11–25.12)	0.43
	Tertiary	25 (15.92)	1.45 (0.79–2.64)	0.23	1.57 (0.79–3.14)	0.20
Bed net use	Yes	25 (14.37)	1	–	1	–
	No	26 (12.62)	0.86 (0.47–1.55)	0.60	1.90 (0.71–6.18)	0.24
Number of net use	≧5	20 (21.05)	1	–	1	–
	< 5	31 (10.88)	0.45 (0.24–0.85)	0.01^*^	0.25 (0.07–0.73)	0.02^*^
Gravidity	≧2	32 (12.03)	1	–	1	–
	< 2	19 (16.67)	1.48 (0.79–2.72)	0.21	1.53 (0.65–3.62)	0.33
Parity	≧3	11 (11.00)	1	–	1	–
	< 3	40 (14.29)	1.35 (0.69–2.88)	0.40	1.09 (0.44–2.84)	0.85
ANC	≧4	36 (12.95)	1	–	1	–
	< 4	15 (14.71)	1.15 (0.59–2.17)	0.66	1.06 (0.50–2.17)	0.87
Gestational age	≧37	36 (10.40)	1	-	1	–
	< 37	15 (44.12)	6.78 (3.14–14.50)	< 0.001^***^	7.79 (3.33–18.39)	< 0.001^***^
Residence	Semi urban	37 (13.36)	1	–	1	–
	Rural	14 (13.59)	1.03 (0.52–1.96)	0.93	1.20 (0.56–2.49)	0.62
malaria in pregnancy	No	36 (13.58)	1	–	1	–
	Yes	15 (13.04)	0.96 (0.49–1.81)	0.91	0.98 (0.47–1.97)	0.96

^*^ Significant association (p < 0.05) with bed net use in pregnancy; *** Indicates significant result (p < 0.05)

In both the bivariate and multivariate models of logistics regression, parturient women were seven times more likely to deliver babies with low birth weights if they had a gestational age < 37 weeks compared to their counterparts with gestational age of 37 weeks or more (aOR = 7.79 (3.33–18.39), p < 0.0001). In the same way, mothers with low birth weight babies at delivery were twice more likely to have used bed nets for less than five months compared to mothers with normal birth weight babies (aOR = 1.53 (0.65–3.62) p = 0.02). All other variables, including reported malaria during pregnancy, were insignificantly associated with low birth weight in different models.

## Discussion

The objective of this survey was to assess the uptake of Intermittent Preventive Treatment (IPT) during pregnancy and any possible relationship with birth weight of newborns and maternal anaemia. Out of the 380 pregnant women participating in the survey, the most represented group was those aged between 24 and 34 years. Results were comparable to those of Mlugu et al*.* in Tanzania [30], where the most represented group was aged between 20 and 34 years, and those of Mutanyi et al*.* in Kenya [31] where the most represented was aged between 24 and 34 years. However, the finding was different from earlier reports of Toure et al*.* in Ivory coast [32] where the most represented women were over 30 years of age, and those of Igboeli et al*.* in Nigeria [33] with women aged between 18 and 34 years. The difference observed between all these studies could be due to the methods used by the authors for grouping participants according to their age.

Uptake of the WHO recommended doses of IPTp was observed in two of every three parturient mothers in the study population. This finding shows uptake to be higher than the observation in Western region of Ghana (47.7%) [34], Tanzania (40.6–52.6%) [35] and in Ghana (42.4%) [36]. This difference might be due to differences in implementation challenges, procurement bottlenecks, geographical variations in the intensity of malaria transmission and irregular compliance to the IPT services by pregnant women.

Among those who received at least one IPTp, more than 40% of them received at least a dose of dihydroartemisinin-piperaquine (DP) or a mix of DP and SP instead of the recommended SP in the local guideline. In addition, contextual factors and wealth index, which is reflected in the educational status of the women could also affect uptake of ≧ 3 IPTp during pregnancy. The majority of women had secondary education and although may be considered knowledgeable of chemopreventive measures in pregnancy, they may not have perceived the risk of malaria infection and its consequences for the mother and new born in this low malaria transmission region. This could have affected uptake of IPTp. However, perceived risk was not explored in this study and therefore this hypothesis needs further exploration. Further, in this study, no microscopic infection with malaria parasite was found in the placental blood although 6 (1.57%) of placental samples were positive for *P. falciparum* after molecular diagnosis by using molecular methods. Several reasons could account for this. It has been shown that the yield of microscopy increases if placental histology is done (impression smears) rather than smears of blood from intervillous space [17]. Thus, histology is a better indicator of malaria parasite status in placenta; which was not performed in this study. Secondly, malaria transmission in Dschang is low and spatially heterogeneous and, therefore, the risk of exposure to placental or peripheral infections at birth is small at population scale despite increased vulnerability of this group. In a similar study published by Apinjoh et al*.* in the South West region of Cameroon [37], and in the Congo by Mbouamboua et al*.* [38] revealed a higher proportion (25% and 16.7%, respectively) of submicroscopic infections detected by PCR. This observation lends support to the difficulty in properly diagnosing malaria in pregnancy and supports the use of chemoprevention to protect the developing fetus and prevent adverse outcomes at birth.

Exposure to malaria in pregnancy is an important factor in predisposing to poor birth outcomes among parturient women. Malaria in pregnancy, irrespective of when it occurs can cause placental malaria infections, placental insufficiency leading to poor birth outcomes including delivery of low birth weight babies and maternal anaemia. In addition, malaria in pregnancy was reported in one in every four parturient women. Although this prevalence was lower than 29.5% found by Kamissoko in Bamako [39], it was higher than the 7.9% and 19% reported by Agyeman et al. in Ghana and Arnaldo et al*.* in Mozambique, respectively [36, 40]. This reported prevalence of malaria in pregnancy in Dschang could be related to low bed net usage due to delays in the distribution of treated bed nets since 2020 (District Health Service, unpublished). This is also corroborated by low bed net coverage observed in the present study. However, associations with reported malaria during pregnancy should be interpreted with some caution as the variable could be associated with recall issues despite the measures taken to prevent this. An association between reported malaria exposure in pregnancy and mild maternal anaemia at birth was found. Although anaemia in pregnancy can also be caused by iron-folate acid deficiency, both parameters are highly associated with malaria infections in pregnancy. The rate of iron deficiency anaemia in a sample of third trimester women was recently shown by Anang [41] to be 6% among third trimester pregnant women. indicating the observation of maternal anaemia in women at delivery could be strongly linked to malaria exposure in pregnancy. However, it would be important to discriminate all independent risk factors associated with maternal anaemia in pregnancy for any meaningful control measure to be designed.

Low birth weight among babies born to women is a serious problem to the development and future outcome of the baby. Malaria can cause low birth weight through several mechanisms including placental inflammation and dysregulated placental development, which has been shown to affect placental vasculogenesis, angiogenesis and nutrient transport. Fetal growth restriction ensues and increased risk of mortality [42, 43]. Analysis showed that 13.9% of newborns had low birth weight. This finding was found to be comparatively higher than 12.1%, 9.7% and 9.6% reported in studies conducted by Aminata et al*.* in Bamako [44], Toure et al*.* in Ivory Coast [32] and in northern Uganda [45, 46]. While microscopic parasite infections in placenta blood of parturient women were not detected, five infections were confirm by PCR from microscopic negative placental samples coming from afebrile women. Sub-microscopic placental parasite infections is not uncommon among women at delivery [37, 38, 47]; and it has been associated with low birth weight in a study in Sudan [48]. Placental impressions smears, associated with more accurate determination of parasite status of the placenta than blood from the intervillous space, were not made.

Taken together, the findings from this study indicated moderately high uptake of ≥ 3 SP in pregnancy in this setting and associated with number of ANC contacts during pregnancy while gestational age at birth < 37 weeks and low bed net coverage predicted low birth weight. Anaemia on the other hand was predicted by reported malaria during pregnancy although among women at delivery, submicroscopic placental parasite infection rate was 1.31% by PCR-based diagnosis. Stock-outs accounted for women taking either SP, sequentially SP and DP or DP for IPT during pregnancy. This study was limited in that other methods of detection, such as placental impression smears were not used to confirm our microscopic observations. However, the implementation of well-known quality assurance measures for light microscopy and application of molecular detection techniques allowed us to conclude on very high-quality readings.

## Conclusion

The present study revealed two-third of women at delivery in the Regional Hospital Annex Dschang took three or more IPT doses in line with WHO recommendations. At least antenatal care contacts independently predicted uptake of 3 or more IPT doses. Similarly, reported malaria in pregnancy predicted maternal anaemia at delivery while gestational age less than 37 weeks and bed net use for less than 5 months in pregnancy independently predicted low infant birth weight in the study sample. Of pregnant women who received at least a single dose of IPTp during that time, 77.2% took either SP alone, DP alone or DP and SP during ANC contacts reflecting possible SP stockout. A low prevalence of submicroscopic placental parasite infections was observed at delivery, all due to *P. falciparum,* and this may reflect overall low transmission of malaria in the area. Modifiable structural and facility based factors should be addressed to improve ANC attendance, bed net use and increased IPT uptake in the community of pregnant women in Dschang.

### Supplementary Information

**Additional file 1.** A summary of reaction conditions for the PCR based diagnosis of malaria using placental blood samples.

## Data Availability

The data generated during the current study are included in the manuscript and any supporting files.

## Abbreviations

ANC: Antenatal consultation
IPT: Intermittent preventive treatment
MIP: Malaria in pregnancy
SP: Sulfadoxine Pyrimethamine
DP: Dihydroartemisinin piperaquine
WHO: World Health Organization
