# Supplementary material for: Uptake of intermittent preventive treatment of malaria in pregnancy and risk factors for maternal anaemia and low birthweight among HIV-negative mothers in Dschang, West region of Cameroon: a cross sectional study
Source: Malar J. 2024 Jan 4;23:6. doi: 10.1186/s12936-023-04816-8 (PMC10768405; doi:10.1186/s12936-023-04816-8)
Supplement: Supplementary file 1 — Additional file 1. A summary of reaction conditions for the PCR based diagnosis of malaria using placental blood samples. [file 12936_2023_4816_MOESM1_ESM.docx]

***Plasmodium* genus *18S* rRNA primers**

rPLU1: 5’-TCA AAG ATT AAG CCA TGC AAG TGA-3’

First PCR (1670 bp)

rPLU5: 5’-CCT GTT GTT GCC TTA AAC TCC-3’

**Ref: (Snounou et al. 2002)**

**PCR Condition:**

94.0 °C 5 min

94.0 °C 30 sec

55.0 °C 30 sec x40

72.0 °C 1 min

72.0 °C 5 min

rPLU3: 5’- TTTTTATAAGGATAACTACGGAAAAGCTGT-3’

Nested PCR (235 bp)

rPLU4: 5’- TACCCGTCATAGCCATGTTAGGCCAATACC-3’

**PCR Condition:**

**Ref: (Snounou et al. 2002)**

94.0 °C 5 min

94.0 °C 30 sec

62.0 °C 30 sec x40

72.0 °C 30 sec

72.0 °C 5 min

***P. falciparum* specific primers**

rFAL1: 5’-TTAAACTGGTTTGGGAAAACCAAATATATT-3’

*P. falciparum* (206 bp) (nested)

rFAL2: 5’- ACACAATGAACTCAATCATGACTACCCGTC-3’

**Ref: (Snounou et al. 2002)**

**PCR Condition:**

94.0 °C 5 min

94.0 °C 30 sec

55.0 °C 1 min x40

72.0 °C 1 min

72.0 °C 5 min
